# The sperm’s tale

**DOI:** 10.1186/2041-2223-5-6

**Published:** 2014-06-18

**Authors:** Mark A Jobling

**Affiliations:** 1 Department of Genetics, University of Leicester, University Road, Leicester LE1 7RH, UK

## 

Copenhagen is as flat as the proverbial pan-cake. A good way to appreciate this is to visit the Rundetaarn, a 35 m tower whose summit is reached by a spiral ramp designed in the 17th century to accommodate a horse and cart carrying books to its elevated library, and in more recent times inviting the competitive attention of puffing middle-aged blokes, and occasional maniacs on unicycles. Squeeze past the other tourists in the ascent of the final staircase, and you are treated to a view stretching out in all directions. On our recent visit we were pleased to see in the distance the televisually iconic Øresund Bridge connecting Sweden and Denmark, but there was not so much as a hint of a hill.

This flatness perhaps goes some way to explain the enormous number of Copenhagen’s pedal-driven vehicles (mostly of the two-wheeled variety), which present a serious hazard to the unwary pedestrian. While you are unlikely to be terrorised by the aggressive lycra-clad warriors typical of London, you are under constant threat from a motley crowd of bicycles, including many with huge low-slung boxes on the front occupied by the cyclist’s family or friends, or some irritatingly stylish furniture items.

**Figure 1:**
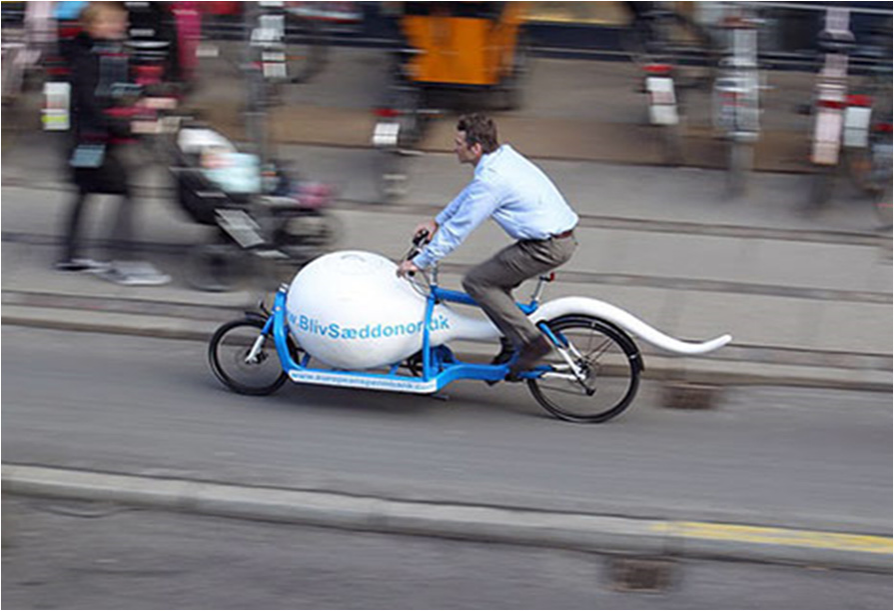
Copenhagen’s Sperm Bike in action.

However, we were disappointed not to encounter the city’s notorious Sperm Bike. This eccentric conveyance (Figure 1) has a blue frame, and instead of a cargo-box a liquid nitrogen tank in the shape of a sperm-head, full of (you guessed it) frozen semen samples. The monster sperm’s tail waves jauntily behind, arcing over the rear wheel like an axonemic mud-guard. This Sperm Bike criss-crosses Copenhagen carrying samples from donors to recipients, and is symptomatic of Denmark’s relaxed attitude to assisted reproductive therapy (ART). Rather as Switzerland has become the UK citizen’s destination of choice for assisted suicide, at the opposite end of life Denmark thrives in ‘fertility tourism’. This is partly because of a 2005 change in UK law, which meant that donors were no longer anonymous. This led to a fall in donations from the reserved Brits, and a sperm shortage. In a newspaper article [1] entitled ‘*The Father’s a Viking’*, a mother-to-be describes her experience of travelling to Copenhagen, and parting with £460; she says she will call the resulting daughter Freya, and comments that her own family are from the north and west of the British Isles, and therefore have Scandinavian blood already. Pity the future population geneticist who tries to understand the history of the Viking migrations.

As a man gets older, his semen volume and sperm count decline [2], though elderly men still father healthy children, as the lively 96-year-old Ramjit Raghav demonstrated in 2012. But a more interesting effect of older fathers is the increased risk of passing mutations to the next generation. Births of children with short-limbed dwarfism (achondroplasia) were shown by Lionel Penrose in the 1950s [3] to become more likely as paternal age increased, and the same was later shown to be true of a suite of other dominantly-inherited genetic diseases. Today, next-generation sequencing can be used to detect *de novo* base substitutions directly by analysing the genomes of parents and their offspring. This shows that the paternal mutation rate is 3.9 times the maternal rate [4], and demonstrates a linear increase of about two new mutations per year due to ageing dads, but not mums.

The widely accepted explanation comes down to the difference between the ways in which eggs and sperm are made - while a woman’s eggs have all been produced by the time she is born, a man goes on producing sperm throughout his life from spermatogonial stem-cells (SSCs), which divide about 23 times a year. Cell division means an opportunity for mistakes in DNA replication, so the errors keep accumulating with age. This explanation is probably right for mutation in general, though for some particular functional mutations, including those that cause achondroplasia, an exponential increase with age points to the selection of ‘selfish’ mutations in the testis that lead to clonal expansion of subsets of SSCs [5]. Because of the age effect, registered sperm donors in the UK must be 41 years old or younger. Furthermore, a rational course of action for young men nervous about the mutational load in their children might be to freeze their sperm for use in later life. Not very romantic, though.

Sperm donors are also limited in the number of donations they can make, though this varies considerably (from 1 to 25) in different countries. The arguments for the appropriate number are based on keeping the likelihood of half-sibling matings among the donor’s progeny the same as that in the general population [6]. However, such matings would only happen if donors were anonymous, and as open-identity of donors becomes the norm, such calculations should become unnecessary. Unhappy incidents have occurred like the case of the Dutch donor who fathered 18 children before discovering he had a late-onset autosomal dominant cerebellar ataxia [7], and these rarities tend to lead to reductions in the per-donor limit, which is unfortunate given the general shortage of sperm samples available for ART.

One genetically influenced trait that sperm donors are unlikely to pass on is male infertility. However, even this trait is now potentially heritable, thanks to intracytoplasmic sperm injection (ICSI). Sperm, either isolated directly from the testis, or from poor-quality semen, are directly injected into the oocyte, thus bypassing the barrier of natural selection. The method has been in use since the early 1990s, and thousands of ICSI babies born. On the whole, the method seems safe, but genetic causes of male infertility, such as Y-chromosomal deletions, are passed on to sons [8], and they in turn will require ICSI if they wish to have children.

Given its current status as a premier ART destination, it is somewhat ironic that Copenhagen is also the original source of apocalyptic rumours about an inexorable fall in sperm-counts through time. An influential paper in 1992 [9] reported a drop of approximately 50% in global measures of sperm-count (and also in semen volume) between 1940 and 1990. There has been much subsequent debate over whether this phenomenon is real, or an artefact of a lack of standardised methods for counting [10]. There has also been a search for a reason for the decline, with theories ranging from environmental chemicals mimicking oestrogen, to the wearing of tight underwear. The latter appears to be true [11] - so bikers (Sperm-Bikers included) beware!
